# Immunological Phenotyping of Mice with a Point Mutation in *Cdk4*

**DOI:** 10.3390/biomedicines11102847

**Published:** 2023-10-20

**Authors:** Mehmet Yabas, Gerard F. Hoyne

**Affiliations:** 1 Department of Immunology, The John Curtin School of Medical Research, The Australian National University, Canberra, ACT 0200, Australia; 2 Department of Immunology, Faculty of Medicine, Malatya Turgut Ozal University, Malatya 44210, Türkiye; 3 School of Health Sciences and Physiotherapy, Faculty of Medicine, Nursing, Midwifery and Health Sciences, University of Notre Dame Australia, Fremantle, WA 6959, Australia; 4 Institute for Respiratory Health, QEII Medical Centre, Nedlands, WA 6009, Australia

**Keywords:** CDK4, T cells, NK cells, immunology

## Abstract

Cyclin-dependent kinases (CDKs) play a crucial role in regulation of the mammalian cell cycle. CDK4 and CDK6 control the G1/S restriction checkpoint through their ability to associate with cyclin D proteins in response to growth factor signals. CDK4 deficiency in mice gives rise to a range of endocrine-specific phenotypes including diabetes, infertility, dwarfism, and atrophy of the anterior pituitary. Although CDK6 deficiency can cause thymic atrophy due to a block in the double-negative (DN) to double-positive (DP) stage of T cell development, there are no overt defects in immune cell development reported for CDK4-deficient mice. Here, we examined the impact of a novel *N*-ethyl-*N*-nitrosourea-induced point mutation in the gene encoding CDK4 on immune cell development. Mutant mice (*Cdk4^wnch/wnch^*) showed normal development and differentiation of major immune cell subsets in the thymus and spleen. Moreover, T cells from *Cdk4^wnch/wnch^* mice exhibited normal cytokine production in response to in vitro stimulation. However, analysis of the mixed bone marrow chimeras revealed that *Cdk4^wnch/wnch^*-derived T cell subsets and NK cells are at a competitive disadvantage compared to *Cdk4^+/+^*-derived cells in the thymus and periphery of recipients. These results suggest a possible role for the CDK4^wnch^ mutation in the development of some immune cells, which only becomes apparent when the *Cdk4^wnch/wnch^* mutant cells are in direct competition with wild-type immune cells in the mixed bone marrow chimera.

## 1. Introduction

During development, cells respond in a coordinated and temporal manner to environmental cues such as the presence of growth factors, but in the absence of survival signals, cells may die via apoptosis or enter a state of senescence. Cyclin-dependent kinases (CDKs) belong to a family of serine threonine kinases and are important for organ development and homoeostasis. CDKs play a crucial role in the regulation of two key checkpoints that control the commitment to cell division. The G1/S transition phase of the cell cycle is the restriction checkpoint that occurs in response to mitogenic signalling [1]. The G1 restriction checkpoint is controlled by CDK4 or the closely related CDK6 which both bind D-type cyclins, while CDK2 binds cyclin E [2]. The G2/M transition phase is regulated by CDK2/cyclin A or cyclin E complexes [2]. The G1 CDK/cyclin complexes phosphorylate a number of key G1 target proteins, such as retinoblastoma protein (pRb). Normally, pRb is in a hypophosphorylated state and acts as a transcriptional repressor by tethering the E2F transcription factor in the cytoplasm [3]. Upon growth factor signalling, pRb becomes phosphorylated on multiple sites controlled by the activity of CDK4/cyclin D and CDK2/cyclin E [4,5]. The phosphorylation of pRb releases E2F to translocate to the nucleus to regulate gene transcription of S phase targets such as cyclins A, E, and DNA polymerase-α [6].

The immune system is comprised of the innate and adaptive immune responses that are composed of distinct cell types that help protect the host from the threat of infection. Innate immune cells sense pathogens within tissues and can coordinate the expansion and differentiation of innate effector cells in an effort to restrict the spread of the pathogen, but also play a critical role in coordinating the adaptive immune responses mediated by lymphocytes. Lymphoid cell development occurs in the bone marrow and thymus, which gives rise to B and T cells, respectively, and control of cell cycle progression is critical for normal developmental pathways. Pre-B and pre-T cells share a similar requirement for clonal proliferative expansion after engagement of the pre-B cell receptor (surrogate IgL^+^ IgH^+^) or pre-T cell receptor (pre-Tα^+^ TCRβ) [7]. These cells proliferate briefly and then exit the cell cycle to rearrange the kappa light chain for B cells or α chain for T cells that lead to expression of a mature antigen receptor. Cyclin D3 has a nonredundant role in regulating proliferation of both pre-B and pre-T cells [8,9,10]. Likewise, the BCR in peripheral mature B lymphocytes induces cell division by upregulating cyclin D2 transcription [11]. Cytokine signalling is important for determining cell fate during T and B cell lymphopoiesis [12,13] and peripheral activation [14,15]. Responsiveness of lymphocytes to cytokines guides proliferation and differentiation into effector cell subsets. CDK4 appears to play an important role in enabling human T cells to respond to IL-2 [16,17,18]. Similarly, mature B cells, in order to progress through S phase of the cell cycle, require the accumulation of CDK2, CDK4, and cyclin D2 [19,20,21].

Although CDK4 is ubiquitously expressed in tissues throughout body, CDK4-deficient mice can survive embryogenesis [22,23], suggesting that CDK4 is dispensable during embryonic development or its loss can be compensated by the other kinases. Two independent CDK4 knockout alleles were generated in mice and both display a unique endocrine phenotype, which is characterized by the loss of pancreatic beta cells leading insulin deficiency and diabetes, dwarfism and infertility [22,23,24,25]. It is believed that CDK2 and CDK6 can provide a compensatory function in the absence of CDK4 for most tissues except for some with endocrine function [24]. Despite the other abnormalities, CDK4 deficiency in mice did not affect the development of major immune cell subsets in the primary and secondary lymphoid tissues with the exception of hypoplastic thymuses and increased CD4^−^CD8^−^ double-negative (DN) T cells in *Cdk4*^−^*^/^*^−^ mice [26].

Here, we investigated the impact of a novel *N*-ethyl-*N*-nitrosourea (ENU)-induced point mutation in the gene encoding CDK4 on immune cell development in vivo. Mutant mice (*Cdk4^wnch/wnch^*) have a mutation on the stop codon, which allows translation of an additional 120 nucleotides, which adds an extra 40 amino acids to the C-terminus of the protein. Similar to CDK4-null mice, *Cdk4^wnch/wnch^* mice have normal immune cell development. However, T cell subsets and NK cells exhibited a competitive disadvantage for survival and/or proliferation in the mixed bone marrow chimeric recipients.

## 2. Materials and Methods

### 2.1. Mice

The *Cdk4^wnch/wnch^* mouse strain was generated through ENU mutagenesis screen at the Australian Phenomics Facility, Australian National University [27,28]. The *Cdk4^wnch/wnch^* strain was backcrossed onto the C57BL/6 background [27,28]. All mice were housed and maintained in specific pathogen-free conditions at the Australian Phenomics Facility and all animal procedures were approved by the Australian National University Animal Ethics and Experimentation Committee on protocols J-IG-31-04 and J-APF-09-07.

### 2.2. Cell Preparation and Flow Cytometry

Single-cell suspensions of cells from the thymus, spleen, and lymph nodes of mice were prepared by passing whole tissues through a 70 μm nylon cell strainer. Live cells in the cell suspensions prepared from the tissues were counted using the trypan blue dye exclusion method and the absolute number of cells was quantified by multiplying the number of live cells by the percentage of the cell population of interest analyzed by flow cytometry. Cells were labelled with mixtures made of the following antibodies; APC Cy7-labelled anti-CD3 (clone 145-2C11, BD, San Jose, CA, USA), Alexa Fluor 700-labelled anti-CD3 (clone 145-2C11, eBioscience, San Diego, CA, USA), APC Cy7-labelled anti-CD4 (clone RM4-5, BD), PE-labelled anti-CD4 (clone RM4-5, BD), PerCP-Cy5.5-labelled anti-CD8a (clone 53-6.7, BD), PE-Cy7-labelled anti-CD8a (clone 53-6.7, Biolegend, San Diego, CA, USA), Pacific Blue-labelled anti-CD44 (clone IM7, Biolegend), APC-labelled anti-CD19 (clone 1D3, BD), Alexa Fluor 405-labelled anti-CD45R/B220 (Invitrogen, Waltham, MA, USA), PerCP-labelled anti-CD45R/B220 (clone RA3-6B2, BD), APC-labelled anti-CD25 (clone PC61, BD), FITC-labelled anti-TCRγδ (clone GL3, BD), FITC-labelled anti-TCRβ (clone H57-597, BD), Alexa Fluor 700-labelled anti-CD45.1 (clone A20, Biolegend), PE-labelled anti-CD45.1 (clone A20, BD), PerCP Cy5.5-labelled anti-CD45.2 (clone 104, BD), APC-labelled anti-NK1.1 (clone PK136, BD), FITC-labelled anti-CD11b (clone M1/70, BD), APC-labelled anti-CD11c (clone HL3, BD), APC Cy7-labelled anti-Gr-1 (clone RB6-8C5, BD), anti-CDK4 (clone DCS-31, Invitrogen), anti-CDK2 (clone OTI2D9, Invitrogen), and anti-Alexa Fluor 488-conjugated goat anti-mouse IgG (A-11001). Cell suspensions labelled with an appropriate mixture of antibodies were incubated at 4 °C in the dark for 30 min. Cells were then washed with FACS buffer, PBS containing 2% bovine serum and 0.1% NaN_3_. Intracellular FoxP3 staining was performed using the eBioscience Foxp3/Transcription Factor Staining Buffer Set according to the manufacturer’s instructions. Flow cytometry was performed on a LSR II (BD) and analyzed using FlowJo 887 software (LLC, Ashland, OR, USA).

### 2.3. Bromodeoxyuridine (BrdU) Labelling

The mice were treated with BrdU (FITC mouse anti-BrdU set, BD) for 3 d and analyzed on day 4 for the uptake of BrdU in different cells using flow cytometry.

### 2.4. Intracellular Cytokine Production

To examine intracellular cytokine production, splenic cells were plated in RPMI 1640 medium supplemented with 10% fetal calf serum, 1% penicillin/streptomycin/L-glutamine, 0.1 μm/mL 2-mercaptoethanol, 1% MEM non-essential amino acids, 1% HEPES, and 1% sodium pyruvate. The cells were incubated in the presence of phorbol myristate acetate (PMA)/ionomycin for 6 h in total, but over the last 2 h of culture, Golgi stop was added. Cells were stained with cell surface antibodies and permeabilized and fixed using the eBioscience Foxp3/Transcription Factor staining Buffer set according to the manufacturer’s instructions prior to the addition of PE-labelled anti-IFNγ (clone XMG1.2, BD) and APC-labelled anti-TNFα (clone MP6-XT22, eBioscience) antibodies. CD4^+^ and CD8^+^ T cells were then analyzed for the production of IFNγ and TNFα by flow cytometry.

### 2.5. Generation of Bone Marrow Chimeras

Bone marrow chimeric mice were generated as previously published [29] by injecting *Cdk4^+/+^* CD45.1/2 cells mixed with *Cdk4^wnch/wnch^* CD45.2 cells into *Cdk4^+/+^* CD45.1 recipients irradiated with a double dose of 450 rads. Donor cells were mixed at a 50:50 ratio and injected intravenously into the tail vein of 6–8-week-old recipients at 200 µL/mouse (2 × 10^6^ cells). Mice were sacrificed approximately 10 weeks after the transplantation for the analysis.

### 2.6. Statistical Analysis

For comparison of the two groups, the unpaired Student’s *t*-test was performed. For all statistical analysis, differences were taken to be significant when *p* < 0.05. All statistical analysis was performed using GraphPad Prism 5 (GraphPad Software, San Diego, CA, USA).

## 3. Results

### 3.1. The Effect of the CDK4^wnch^ Mutation on Immune Cell Subsets

**Figure 1:**
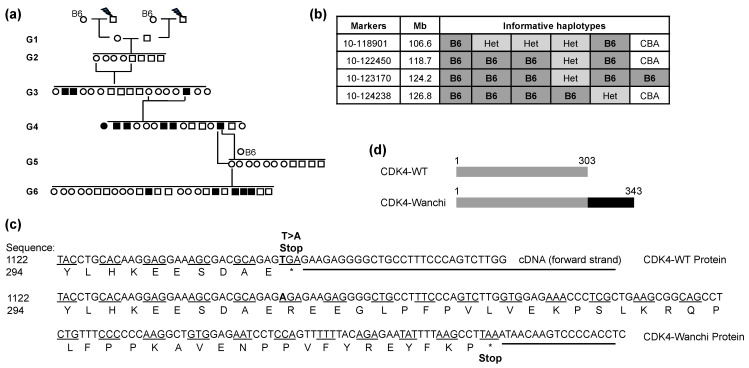
An ENU-induced point mutation on the stop codon of *Cdk4* in the Wanchi (*Cdk4^wnch/wnch^*) mouse strain. (**a**) A pedigree analysis of the ENU-induced Wanchi strain over six generations. Square symbols represent males, and circles represent females. Filled shapes represent diabetic animals, and clear symbols had no diabetes. (**b**) Meiotic mapping of the CDK4^wnch^ mutation using B6 x CBA F2 DNA samples to a 2.6 Mbp region on the distal end of chromosome 10. (**c**) Nucleotide sequence of the C-terminal region of the *Cdk4* gene. Top sequence is derived from *Cdk4^+/+^* mice, and the bottom nucleotide sequence is that of *Cdk4^wnch/wnch^* mice. The amino acid sequence stops in the wild-type protein at 303 amino, but due to a mutation in the *Cdk4^wnch/wnch^* mice, the stop codon converts to arginine and allows transcription of an additional 120 nucleotides (bold sequence) before the next in-frame stop codon. * denotes stop codons. (**d**) Diagrammatic representation of the CDK4 protein from *Cdk4^+/+^* and *Cdk4^wnch/wnch^* mice.

During the analysis of ENU-treated pedigrees, a mouse strain was identified that developed diabetes (Hoyne et al. in preparation). Diabetes susceptibility was inherited as a recessive trait (Figure 1a) and the causative mutation in the Wanchi strain was mapped to the distal region of chromosome 10 and localized to a 2.6 Mbp interval, which contained ~34 genes (Figure 1b). A potential candidate gene in the interval was *Cdk4*, and all of the exons were sequenced and a T to A substitution was identified in the stop codon of *Cdk4* in exon 8, leading to a stop codon to arginine change (Figure 1c). The CDK4^wnch^ mutation disrupts the normal stop codon, allowing for the transcription of an additional 120 nucleotides before the next in-frame stop codon (Figure 1c,d). The mutation adds an additional 40 amino acids to the C-terminal tail, which is expected to increase its molecular weight (Figure 1c,d).

**Figure 2:**
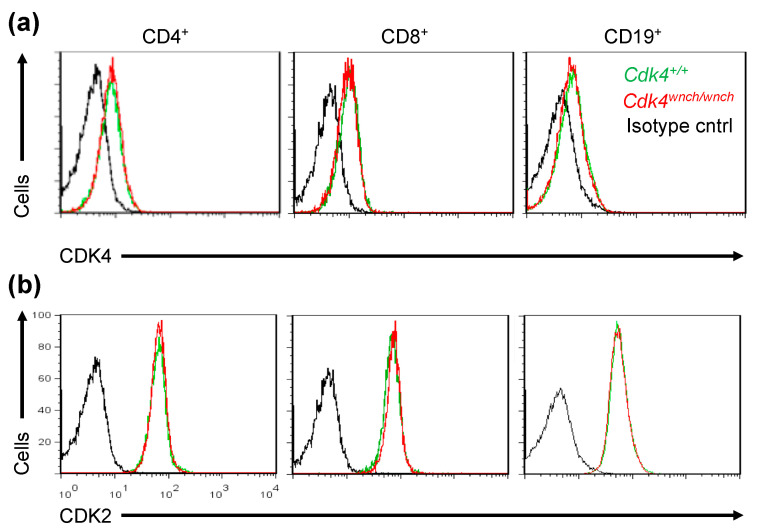
The CDK4^wnch^ mutation does not affect expression of G1-dependent CDKs. Representative overlay flow cytometry histograms of (**a**) CDK4 and (**b**) CDK2 expression in splenic CD4^+^ T cells, CD8^+^ T cells, and CD19^+^ B cells from *Cdk4^+/+^* (green line) and *Cdk4^wnch/wnch^* mice (red line). Isotype control staining is shown with a black line. Data are representative of four mice per genotype.

We first examined the expression of CDK4 and CDK2 by flow cytometry in splenic CD4^+^, CD8^+^ T cells, and CD19^+^ B cells from *Cdk4^+/+^* or *Cdk4^wnch/wnch^* mice. Analysis revealed that there was no difference in the level of expression of either CDK4 or CDK2 proteins in *Cdk4^+/+^* or *Cdk4^wnch/wnch^* lymphocytes (Figure 2a,b).

**Figure 3:**
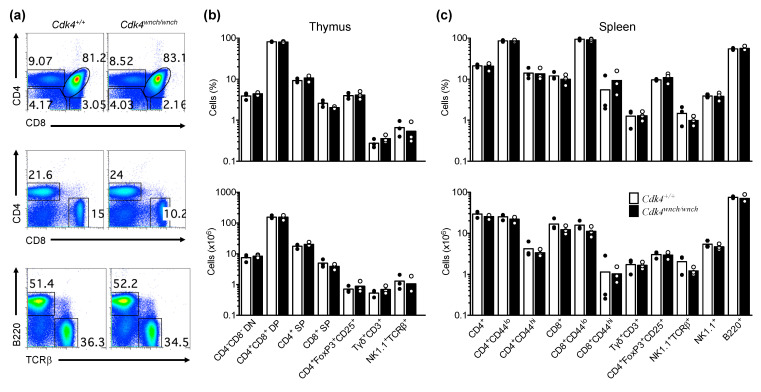
Lymphocyte development proceeds normally in *Cdk4^wnch/wnch^* mice. (**a**) Representative flow cytometric plots of CD4 versus CD8 staining of thymocytes (top panel) and splenocytes (middle panel) and B220 versus TCRβ (bottom panel) staining in the spleen of *Cdk4^+/+^* and *Cdk4^wnch/wnch^* mice. Graphs show the percentage and absolute number of different immune cell subsets (**b**) in the thymus and (**c**) in the spleen of *Cdk4^+/+^* and *Cdk4^wnch/wnch^* mice identified by flow cytometry. Data are representative of four independent experiments and show the mean with three mice per genotype. Each symbol represents an individual mouse.

To determine if the CDK4^wnch^ mutation has any effect on immune cell development, we used flow cytometry to examine different immune cell subsets in the primary and secondary lymphoid tissues during postnatal development. T cells undergo a well-characterized series of developmental steps in the thymus from DN to double-positive (DP) to give rise to mature CD4^+^ or CD8^+^ T cells as a result of negative and positive selection [30,31]. Analysis of T cell subsets in the thymus of *Cdk4^+/+^* and *Cdk4^wnch/wnch^* mice showed that CD4^+^ and CD8^+^ T cell differentiation was normal throughout postnatal life (Figure 3a,b). Likewise, the frequency and number of mature CD4^+^ and CD8^+^ T cells in the periphery of *Cdk4^wnch/wnch^* mice was similar to that of *Cdk4^+/+^* mice (Figure 3a,c). Further analysis of T cells subsets using the CD44 surface marker revealed a normal distribution of naïve (CD44^lo^) and memory (CD44^hi^) T cells in the spleen of *Cdk4^wnch/wnch^* compared with *Cdk4^+/+^* mice (Figure 3a,c).

The TCRγδ^+^ cells have important roles in the immune system, such as tumour surveillance and mucosal regulation, and their primary development occurs in the thymus [32]. We wanted to examine if the CDK4^wnch^ mutation might affect the homeostasis of TCRγδ^+^ cells. The analysis of thymus and spleen revealed that the percentage and number of TCRγδ^+^ cells in *Cdk4^wnch/wnch^* mice were comparable to those observed in *Cdk4^+/+^* mice (Figure 3b,c).

Thymus-derived CD4^+^FoxP3^+^CD25^+^ regulatory T (Treg) cells are a specialized subpopulation of CD4^+^ T cells that arise during the Tαβ cell differentiation in the thymus [33]. Treg cells have important roles in the maintenance of self-tolerance and regulating adaptive immune responses [33]. We evaluated the thymic and peripheral Treg cells and found that the frequency and absolute number of Treg cells are not significantly different between *Cdk4^+/+^* and *Cdk4^wnch/wnch^* mice in the thymus and spleen (Figure 3b,c). This finding is consistent with the observation that *Cdk4^wnch/wnch^* mice do not develop spontaneous autoimmunity. The development of diabetes in the *Cdk4^wnch/wnch^* mice is caused by an intrinsic defect in pancreatic beta cells and is not associated with immune destruction of the islet tissue (Hoyne et al. in preparation).

Similarly, analysis of NK1.1^+^TCRβ^+^ NKT cells, NK1.1^+^ NK cells, and B220^+^ B cell populations revealed that both *Cdk4^+/+^* and *Cdk4^wnch/wnch^* mice have an essentially comparable percentage and absolute number of those cells in the thymus and spleen (Figure 3a–c). Taken together, these findings suggest that the CDK4^wnch^ mutation does not affect the differentiation or accumulation of major immune cell subsets in mice.

### 3.2. Proliferation Capacity of Cdk4^wnch/wnch^ Cells Is Normal

**Figure 4:**
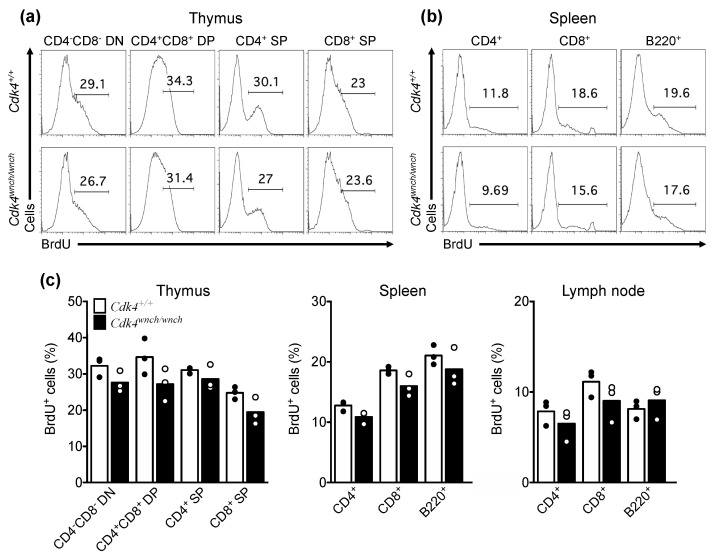
Homeostatic proliferation of lymphocyte subsets is normal in *Cdk4^wnch/wnch^* mice. *Cdk4^+/+^*and *Cdk4^wnch/wnch^* mice were injected with BrdU on 3 consecutive days and cells from the thymus, spleen, and lymph nodes were stained with antibodies against CD4 and CD8 to identify T cells and B220 to identify B cells. Representative flow cytometric histogram plots of BrdU staining of (**a**) thymocytes and (**b**) splenic cells of *Cdk4^+/+^* and *Cdk4^wnch/wnch^* mice. (**c**) Graphs show the percentage of BrdU^+^ cells in different cell subsets in the thymus, spleen, and lymph nodes of *Cdk4^+/+^*and *Cdk4^wnch/wnch^* mice identified by flow cytometry. Data are from one experiment and show the mean with three mice per genotype. Each symbol represents an individual mouse.

Given that the CDK4^wnch^ mutation did not affect the differentiation of immune cell subsets and that CDK4 plays a critical role in the G1/S checkpoint in cell cycle progression, we next wanted to test if the mutation controls the ability of cells to proliferate in vivo. To test this, *Cdk4^+/+^* and *Cdk4^wnch/wnch^* mice were administered with BrdU for 3 days and the uptake of BrdU on day 4 was analyzed by flow cytometry using cells from the thymus, spleen, and lymph node. Although we observed a trend toward a reduction in proliferation capacity of T cell subsets in the thymus and mature T and B cells in the spleen and lymph node of *Cdk4^wnch/wnch^* animals detected by BrdU^+^ cells, there was no significant difference between the groups (Figure 4a–c). This is consistent with normal in vitro proliferation ability of *Cdk4^−^^/^^−^* thymocytes in response to IL-2 and *Cdk4^−^^/^^−^* splenocytes in response to CD3 [26]. These findings suggest that the CDK4^wnch^ mutation has no effect on the homeostatic proliferation of lymphocytes in vivo, which is consistent with normal cell development in intact *Cdk4^wnch/wnch^* mice.

### 3.3. Cytokine Production by Cdk4^wnch/wnch^ T Cells Is Normal

**Figure 5:**
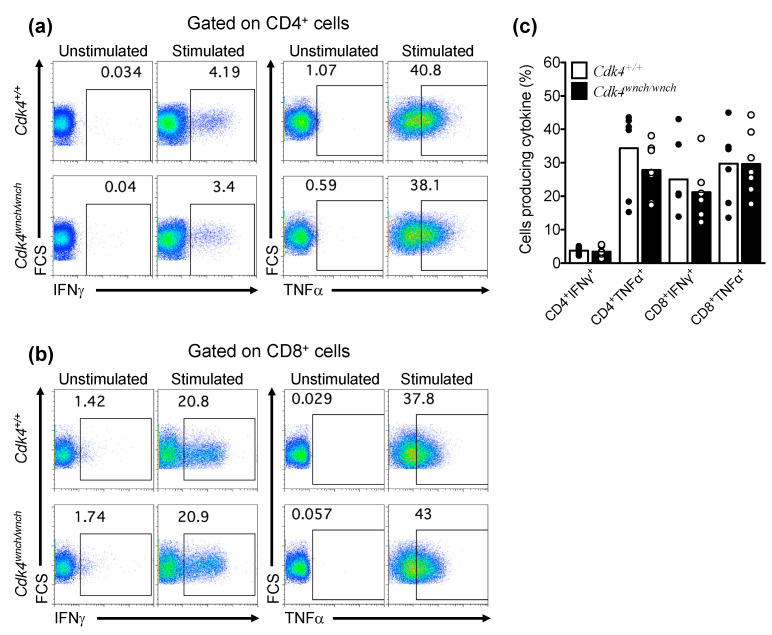
The CDK4^wnch^ mutation does not affect cytokine production by CD4^+^ and CD8^+^ T cells. Splenic cells from *Cdk4^+/+^* and *Cdk4^wnch/wnch^* mice were stimulated with PMA/ionomycin for 6 h and stained for intracellular cytokines. Representative flow cytometric dot plots of IFNγ or TNFα staining of unstimulated or stimulated cells of (**a**) CD4^+^ T cells and (**b**) CD8^+^ T cells. (**c**) Graph shows the percentage of cells producing IFNγ or TNFα from *Cdk4^+/+^* and *Cdk4^wnch/wnch^* mice. Data show the mean with seven mice per genotype analyzed in two different experiments. Each symbol represents an individual mouse.

In response to TCR and costimulatory signals, T cells can proliferate and differentiate into distinct T cell subsets that display signature cytokines. We next tested the ability of peripheral T cell subsets to produce cytokines in response to in vitro stimulus. Splenic cells were cultured in the presence of PMA/ionomycin and Golgi stop, and analyzed for the production of intracellular cytokines in both CD4^+^ and CD8^+^ T cells. We found that CD4^+^ T cells from *Cdk4^wnch/wnch^* mice produced a normal frequency of IFNγ and TNFα cytokines compared to cells of *Cdk4^+/+^* animals (Figure 5a,c). Similarly, the CDK4^wnch^ mutation did not affect the ability of CD8^+^ T cells to produce IFNγ and TNFα in response to in vitro stimulation (Figure 5b,c), indicating that the CDK4^wnch^ mutation does not impede the production of effector cytokines by activated peripheral T cells. This observation recapitulates the findings that splenocytes from *Cdk4^−^^/^^−^* animals demonstrated similar cytokine secretion profiles in response to CD3 stimulation compared to wild-type mice [26].

### 3.4. Reduced Cdk4^wnch/wnch^-Derived T Cell Subsets in Mixed Bone Marrow Chimeric Recipients

**Figure 6:**
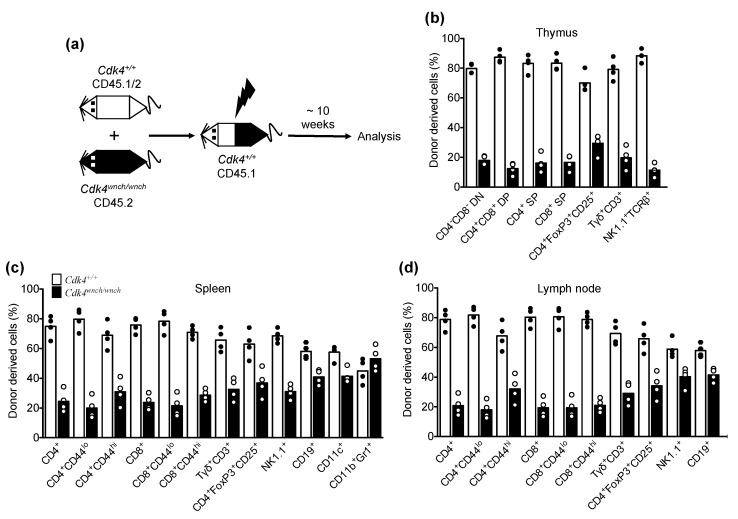
Defects in homeostatic accumulation of *Cdk4^wnch/wnch^*-derived cells were revealed in mixed bone marrow chimeras. (**a**) Experimental approach to test if the CDK4^wnch^ mutation allele impacts lymphocyte development in competitive bone marrow chimeras. Bone marrow chimeric mice were generated by the injection of bone marrow cells from *Cdk4^+/+^* (CD45.1/2) or *Cdk4^wnch/wnch^* (CD45.2) animals mixed at a 1:1 ratio and injected into irradiated *Cdk4^+/+^* (CD45.1) recipients. Approximately ten weeks after reconstitution, the thymus, spleen, and lymph nodes of recipient mice were analyzed by flow cytometry. (**b**–**d**) Graphs show the percentage of *Cdk4^+/+^*- and *Cdk4^wnch/wnch^*-derived cells in the recipient mice. Data are from one experiment and show the mean with four mice. Each symbol represents an individual recipient.

Given the accumulation of T and B cells in the thymus and periphery was normal in *Cdk4^wnch/wnch^* mice, we wanted to investigate the possibility that homeostatic effects were masking a role for the CDK4^wnch^ mutation in T and B cells. To address this point, we generated mixed chimeras using bone marrow cells from mice with different allotypes of the cell surface marker CD45. Bone marrow cells were harvested from CD45.1/2 *Cdk4^+/+^* or CD45.2 *Cdk4^wnch/wnch^* mice. The isolated cells were counted and mixed at a 1:1 ratio and injected into sublethally irradiated CD45.1 *Cdk4^+/+^* recipients (Figure 6a). The two different donor cell populations within the chimeric recipients could be distinguished on the basis of a different expression of CD45 alleles using flow cytometry. The recipient mice were analyzed approximately ten weeks after reconstitution and the results reveal that the percentage of *Cdk4^wnch/wnch^*-derived T cell subsets including CD4^+^, CD8^+^, TCRγδ^+^, and NK1.1^+^TCRβ^+^ NKT cell in the thymus, spleen, and lymph node were all reduced compared to *Cdk4^+/+^*-derived cells (Figure 6b–d). The percentage of *Cdk4^wnch/wnch^*-derived NK1.1^+^ cells in chimeric recipients also appeared to be reduced in the spleen, but less so in the lymph node (Figure 6c,d). The reduction in the percentage of *Cdk4^wnch/wnch^*-derived cell subsets could not possibly be explained by injection of unequal bone marrow cells, as the percentage of *Cdk4^wnch/wnch^*-derived CD19^+^ B and CD11c^+^ dendritic cells was only slightly reduced compared to *Cdk4^+/+^*-derived B cells in the periphery of recipient mice (Figure 6c,d), and there was no difference in the accumulation of *Cdk4^wnch/wnch^*-derived CD11b^+^Gr1^+^ granulocytes compared to *Cdk4^+/+^*-derived cells in the spleen of recipient mice (Figure 6c). Taken together, these data suggest that the CDK4^wnch^ mutation acts in a cell-autonomous manner to regulate homeostatic proliferation and/or survival of T cell subsets and NK cells in vivo, whereas other immune cell lineages are less impacted by the CDK4^wnch^ mutation.

## 4. Discussion

The cyclin-dependent kinases CDK4 and CDK6 play an important role at the restriction checkpoint that guides the G1/S transition of the mammalian cell cycle. The binding of CDKs to D-type cyclins (e.g., cyclin D3) is critical for the development of immature T and B cells [7,8,11]. In addition, CDK/cyclin D signalling mediates cytokine responsiveness of mature T and B lymphocytes [16,17,34,35]. In this study, we wanted to determine if an ENU-induced point mutation in murine *Cdk4* could impact immune cell development and/or function. We found that the CDK4^wnch^ mutation did not affect lymphoid cell differentiation or the homeostatic proliferation of major immune cell subsets in mice. The mutation had also no effect on cytokine production by mature CD4^+^ or CD8^+^ T cells in response to mitogen stimulation. However, in the mixed bone marrow chimeras, there was a reduction in *Cdk4^wnch/wnch^*-derived T cell subsets and NK cells compared to *Cdk4^+/+^*-derived cells. The CDK4^wnch^ mutation had a modest effect on the peripheral B cells and did not affect granulocytes in the mixed bone marrow chimeric recipients. These results suggest that the CDK4^wnch^ mutation might have a role in T cells and NK cells at the stage of development or survival, which is more obvious when CDK4 mutant cells must compete with control cells in vivo.

Previous studies showed that *Cdk4^−^^/^^−^* and *Cdk2^−^^/^^−^* mice display no major defects in T or B cell development [26,36]. *Cdk4^−^^/^^−^* mice had hypoplastic thymuses with decreased total thymocyte cell numbers and increased DN cells, but the ability of cells from *Cdk4^−^^/^^−^* animals to proliferate and produce cytokines was similar to those in wild-type mice [26]. *Cdk6^−^^/^^−^* mice displayed thymic atrophy with a significant reduction in the number of DN and DP cells, while the proportion of mature single-positive CD4^+^ and CD8^+^ T cells were increased [37]. However, there was no reported effect on B cell development in *Cdk6^−^^/^^−^* mice [37]. In addition, CDK6 deficiency protected mice from AKT-driven thymic lymphoma [37]. Cyclin D3 is a major target downstream of the pre-TCR signalling pathway in immature thymocytes to drive expansion of DN3, DN4, and immature single-positive cells to form DP cells [10]. Cyclin D3-deficient mice display a similar blockade of thymocyte development with a reduction in the number of DP cells. Cyclin D3 deficiency can protect mice from T cell leukaemia [10]. These findings suggest a strict requirement for CDK6 and cyclin D3 in early T cell development, while other CDKs are more dispensable. Cyclin D3 is also involved in the development of immature pre-B cells downstream of both cytokine and pre-BCR signalling to allow the expansion of pre-B cells that successfully rearranged the BCR heavy chains [8,9,10]. Again, CDK proteins appear redundant for B cell development. These observations are consistent with our results that the CDK4^wnch^ mutation had no effect on lymphocyte development in *Cdk4^wnch/wnch^* animals in vivo.

The common cytokine receptor gamma chain (γ_c_) is a critical component of the receptors for IL-2, IL-4, IL-7, IL-9, IL-15, and IL-21 that guide the survival, proliferation, and/or differentiation of lymphocytes in the immune system [38]. IL-7 signalling is critical for the early stages of T and B cell development. Homeostatic proliferation of mature naïve lymphocytes requires two signals, one through TCR recognition of self-peptide MHC complexes and the other by IL-7/IL-7R signalling [39,40,41,42]. Memory CD4^+^ and CD8^+^ T cells on the other hand are regulated via distinct mechanisms. CD8^+^ memory T cells can utilize either IL-7 or IL-15, whereas CD4^+^ memory cells do not rely on γ_c_ cytokines [43]. The reconstitution of the peripheral immune system of bone marrow chimeric animals relies on successful homeostatic proliferation of mature cells to fill distinct cellular niches. The CDK4^wnch^ mutation in the mixed bone marrow chimeric recipients did not affect the differentiation of most immune cell subsets, with an exception where there was a significant reduction in the proportion of *Cdk4^wnch/wnch^*-derived T cell subsets and NK cells not only in the thymus, but also within secondary lymphoid tissues. The differentiation of NK cells in vivo is linked to IL-2Rβ expression, which means IL-2 and/or IL-15 can direct their survival and expansion in vivo [44,45]. Thus, the phenotype of *Cdk4^wnch/wnch^* cells in the mixed bone marrow chimeric recipients might be related to cytokine signalling in a competitive environment that is affected by the CDK4^wnch^ mutation.

A clear dichotomy exists in the findings observed for T cells and NK cells between the intact *Cdk4^wnch/wnch^* mice and the *Cdk4^wnch/wnch^*-derived cells in the mixed bone marrow chimeras. Early thymic progenitors within the DN1 (CD44^+^CD25^−^) stage retain a level of multi-potency that can give rise to T cells, NK cells, B cells, and thymic dendritic cells [46]. The ability to generate NK cells and B cells is lost once cells differentiate to the DN2 (CD44^+^CD25^+^) pro-T cell stage. The early stages of T cell development from DN1 to the DN3 stage are guided by cytokine responsiveness and c-kit expression prior to the pre-TCR checkpoint at DN3 [47]. IL-7 is likely the growth factor that controls the G1/S transition in lymphocyte precursors, whereas IL-7 and c-kit ligand together help sustain proliferation as first illustrated in B cell precursors [48]. Beyond the pre-TCR checkpoint, the expansion of immature thymocytes becomes cytokine independent and is directed by the pre-TCR and cells begin to express CDK2, and cyclins A and B to ready them for DNA synthesis [49,50]. The pre-T cell population gives rise to mature CD4^+^ and CD8^+^ TCRαβ^+^ cells and TCRγδ^+^ cells. Thus, any constraint on T cell development prior to DN3 would likely impact on the downstream development of both T cell lineages.

In the thymus of the mixed chimeras, *Cdk4^wnch/wnch^*-derived DN cells were significantly reduced, which suggests that the CDK4^wnch^ mutation compromised the early proliferative expansion of *Cdk4^wnch/wnch^*-derived thymic progenitors to IL-7. In competition with *Cdk4^+/+^* thymic progenitors, *Cdk4^wnch/wnch^* cells were unable to match the proliferative expansion of *Cdk4^+/+^* cells, and *Cdk4^wnch/wnch^* cells were outcompeted during subsequent phases of T cell development. However, *Cdk4^wnch/wnch^*-derived cells must be capable of some proliferative expansion, as mutant cells would not be represented in latter stages of T cell development. Beyond the pre-TCRα checkpoint thymocytes may rely on the activity of CDK6 and CDK2. The reduced thymic output of *Cdk4^wnch/wnch^* T cells was maintained in the peripheral secondary lymphoid tissues. T cell and NK cell emigrants within the mixed bone marrow chimeras would encounter a lymphocyte-deficient environment that would promote homeostatic proliferation in response to IL-7 and IL-15 mediated through γ_c_ signalling [43]. Once immune cells proliferate to fill their cellular niche, naïve T cells rarely divide, while memory T cells would undergo only intermittent cell division [51,52,53]. Therefore, a reduction in the proportion of *Cdk4^wnch/wnch^*-derived T cells and NK cells in the secondary lymphoid tissues could also be due to a defect in the ability of mutant cells to undergo homeostatic proliferation in a competitive situation.

While there were similarities in immune phenotypes between *Cdk4^−^^/^^−^* and *Cdk4^wnch/wnch^* mice (including largely normal development of major immune cell subsets, ability of proliferation, and cytokine secretion) [26], previous studies on the immune function of CDK4-deficient mice never incorporated the analysis of mixed bone marrow chimeras. As revealed here, these experiments highlighted a possible role for CDK4 during the early stages of thymocyte development that was previously overlooked when studies were restricted to the analysis of intact CDK4-deficient mice or chimeric recipients that received only *Cdk4^−^^/^^−^* cells [26]. It was surprising that the CDK4^wnch^ mutation did not have a more pronounced effect on the accumulation of B cells in mixed bone marrow chimeras as the development of pro-B cells involves a similar cytokine-dependent growth stage prior to the pre-B cell stage that relies on IL-7R/γ_c_ signalling [54]. There was only a modest reduction in *Cdk4^wnch/wnch^*-derived mature B cells compared that observed for T cells. Perhaps the homeostatic proliferation of B cells can utilise additional growth factors (e.g., BAFF, April, IL-6, and IL-16) that do not rely on γ_c_ chain signalling [15].

In conclusion, the results of this study highlight a crucial effect of the CDK4^wnch^ mutation on early stages of T cell and NK cell development, which is prominent in the mixed bone marrow chimeras. It is possible that CDK4 is required during the earliest phase of DN1 to DN3 in response to cytokine-dependent growth in a competitive environment, while CDK6 is required to drive expansion downstream of thymocytes from the pre-TCR signalling to direct formation of DP cells prior to their selection into mature single-positive cells. A limitation of this study is that it was restricted to the analysis of an ENU-induced *Cdk4^wnch/wnch^* allele in mice, and future studies are needed to define the biochemical and molecular consequences of the CDK4^wnch^ mutation in immune cell subsets. Although a similar mutant allele was not identified yet in humans, we do know that the CDK4^wnch^ mutation in mice causes the same spectrum of endocrine and dwarf phenotypes observed with *Cdk4^−^^/^^−^* alleles.

## Data Availability

Not applicable.
